# Routine histopathology of gallbladder after elective cholecystectomy for gallstones: waste of resources or a justified act?

**DOI:** 10.1186/1471-2482-13-26

**Published:** 2013-07-08

**Authors:** Faisal G Siddiqui, Ahmer A Memon, Arshad H Abro, Nazeer A Sasoli, Lubna Ahmad

**Affiliations:** 1 Department of Surgery, Liaquat University of Medical & Health Sciences, Jamshoro 71000, Pakistan; 2 Department of Gynea & Obs, Liaquat University of Medical & Health Sciences, Jamshoro 71000, Pakistan

**Keywords:** Gallbladder malignancy, Cholelithiasis, Cholecystectomy

## Abstract

**Background:**

Selective approach for sending cholecystectomy specimens for histopathology results in missing discrete pathologies such as premalignant benign lesions such as porcelain gallbladder, carcinoma-in-situ, and early carcinomas. To avoid such blunders therefore, every cholecystectomy specimen should be routinely examined histologically. Unfortunately, the practice of discarding gallbladder specimen is standard in most tertiary care hospitals of Pakistan including the primary investigators’ own institution. This study was conducted to assess the feasibility or otherwise of performing histopathology in every specimen of gallbladder.

**Methods:**

This cohort study included 220 patients with gallstones for cholecystectomy. All cases with known secondaries from gallbladder, local invasion from other viscera, traumatic rupture of gallbladder, gross malignancy of gallbladder found during surgery was excluded from the study. Laparoscopic cholecystectomy was performed in majority of cases except in those cases where anatomical distortion and dense adhesions prevented laparoscopy. All gallbladder specimens were sent for histopathology, irrespective of their gross appearance.

**Results:**

Over a period of two years, 220 patients with symptomatic gallstones were admitted for cholecystectomy. Most of the patients were females (88%). Ninety two per cent patients presented with upper abdominal pain of varying duration. All specimens were sent for histopathology. Two hundred and three of the specimens showed evidence chronic cholecystitis, 7 acute cholecystitis with mucocele, 3 acute cholecystitis with empyema and one chronic cholecystitis associated with poly. Six gallbladders (2.8%) showed adenocarcinoma of varying differentiation along with cholelithiasis.

**Conclusion:**

The histopathological spectrum of gallbladder is extremely variable. Incidental diagnosis of carcinoma gall bladder is not rare; if the protocol of routine histopathology of all gallbladder specimens is not followed, subclinical malignancies would fail to be identified with disastrous results. We strongly recommend routine histopathology of all cholecystectomy specimens.

## Background

Discarding gallbladder specimens without histopathological analysis is best avoided. Selective approach for sending these specimens to the laboratory results in missing discrete pathologies like premalignant benign lesions such as porcelain gallbladder, carcinoma-in-situ and early carcinomas [1,2]. Early carcinoma of gallbladder notoriously remains undiagnosed without histopathology as it neither produces clinical symptoms or signs nor provides any clues on ultrasound assessment. Cholecystectomy performed with provisional diagnosis of benign diseases based on clinical, ultrasonological and computerized tomographic scanning misses a significant number of early malignant lesions of gallbladder. To avoid such blunders with bad consequences, therefore, every cholecystectomy specimen should be routinely examined histologically [3].

Carcinoma gallbladder carries one of the worst of cancer mortality. It is a frequent underlying pathology in patients undergoing surgical intervention for chronic cholecystitis with cholelithiasis [4]; long standing chronic inflammation by gallstones is considered an important etiological role in carcinogenesis. The incidence of carcinoma gallbladder associated with gallstones varies from 0.3 to 12 per cent [5,6]. Histopathological analysis is therefore mandatory for diagnosis of early carcinomas. Good prognosis is anticipated in patients with gallbladder carcinoma discovered as an incidental finding in stage I disease [5].

The practice of discarding gallbladder specimen is standard in most tertiary care hospitals of Pakistan including the primary investigator’s own institution on the pretext that “surgeon knows best which gallbladder is to be sent to laboratory”. Histopathology is restricted to only those specimens, which show gross abnormalities. This practice is based on the assumption that gallbladder carcinoma is always associated with macroscopic abnormalities. At the same time, this selective approach is justified by claiming that it reduces patient’s financial liabilities and pathologist’s workload. This contradicts to the worldwide practice where gallbladder specimen is invariably sent for histological analysis for the sole purpose of identifying discrete carcinoma in early stage.

This study was conducted to assess the feasibility or otherwise of performing histopathology in every specimen of gallbladder. This would ensure picking of discrete carcinoma of gallbladder, hidden to both the human eyes and touch, which in turn will assist in decreasing mortality rate.

## Methods

This cohort study was carried out at the Department of Surgery, Liaquat University Hospital, Hyderabad/Jamshoro, Pakistan, over a period of two years from March 2010 to February 2012. The study was designed in compliance with the Helsinki Declaration and a formal approval for the study was obtained from the institutional Ethics Review Committee (ERC), Liaquat University of Medical & Health Sciences.

The study consisted of 220 patients with acute or chronic cholecystitis secondary to gallstones admitted through the outpatient Department. Non-probability convenient sampling technique was used. Written, informed consent was taken from all the participants and/or their next of kin where so applied.

Patients with evidence of carcinoma gallbladder, on clinical grounds and confirmed on ultrasonography and/or CT scan were excluded. Gallbladders showing gross abnormalities suggestive of localized or infiltrative malignancy during surgery were excluded. Detailed history and thorough clinical examination of the patients was done with special attention to the right hypochondrium for preoperative assessment of palpable mass. Systemic review was done to see any co-morbidity. Baseline and specific investigations especially ultrasonography of abdomen was done in all patients. Laparoscopic cholecystectomy was performed in all cases but had to be converted to open procedure in few cases where anatomical distortion and dense adhesions prevented any further progress laparoscopically. All gallbladder specimens, including those with no obvious gross abnormality, were sent for histopathology.

A predesigned proforma was used to put down the information gathered. Data were analyzed using SPSS version 17.0.

## Results

Over a period of two years, two hundred and twenty patients with symptomatic gallstones were admitted for cholecystectomy. There were 193 females and 27 males with a male to female ratio of 1:7. The age ranged from 19 to 80 years with the mean age of 32.3 ± 5.3 years (Table 1). Majority of patients (92%) presented with upper abdominal pain of varying duration. Other symptoms are depicted in Table 2.

**Table 1:** Age distribution*

**Age of patients(years)**	**No.of Patients(n=220)**	**%**
**19**-**30**	54	24.6
**31**-**40**	61	27.8
**41**-**50**	59	26.8
**51**-**60**	34	15.4
**61**-**70**	10	4.5
**71**-**80**	02	0.9

*Mean age 32.25±5.3 years.

**Table 2:** Presenting symptoms

**Symptoms**	**No.of Patients(n=220)**	**%**
Pain in Upper abdomen	201	91.4
Intolerance to fatty food	134	61
Nausea and/or vomiting	44	20
Mass in right hypochondrium	5	2.3

All 220 gallbladders were palpated and were opened per-operatively for any focal or diffuse thickening of the gallbladder wall, a raised mucosal plaque, polypoid growth or an infiltrating grey white mass. The specimens were then sent for histopathology. Two hundred and three of the specimens showed evidence chronic cholecystitis, 7 acute cholecystitis with mucocele, 3 acute cholecystitis with empyema and one incidental associated polyp. Six gallbladders (2.8%) showed evidence of adenocarcinoma of varying differentiation along with cholelithiasis, (Table 3).

**Table 3:** Histopathological report (n=220)

**Histopathology**	**Male**	**Female**	**%**
Chronic Cholecystitis	18	185	92.3
Acute cholecystitis with mucocele	5	2	3.2
Acute cholecystitis with empyema	2	1	1.3
Polyp	0	1	0.5
Malignancy	2	4	2.7
**Total**	**27**	**203**	**100**

There were six incidental carcinomas with no gross abnormalities in this series. Subsequent staging revealed three adenocarcinomas in stage T1b, two in stages T2 and one in T3 stage. Three patients with T1 tumors did not undergo any further procedure; three patients with advanced stages (T2 and T3) underwent revision surgery with resection of liver segments 4b and 5 (T2) and right hepatectomy (T3) along with lymphadenectomy. Port site rim of about 2 cms were also excised and sent for histopathology and was found to be free of tumor seedling in all three cases.

## Discussion

In this study, females outnumbered males with male to female ratio of 1:7. Female predominance is also reported by similar studies [7,8]. The mean age 32.25±5.3 years ranging from 19 to 80 years, slightly higher than that reported in other studies [9].

Over ninety one per cent patients presented with pain upper abdomen, a number significantly lower than that reported by Laghari et al [10]. where all patients had upper abdominal pain. None of the patients in our study had any evidence of malignancy either clinically or on ultrasound examination.

The most common histopathological finding in our study was chronic cholecystitis; 203 (92.3%) specimens were reported as chronic inflammation with mucosal ulceration, denudation, metaplasia to dysplasia and wall infiltration by chronic inflammatory cells like neutrophils, macrophages, plasma cells and varying degrees of fibrosis. A similar study by Memon [11] also reports chronic cholecystitis as major histopathological finding, identified in 64.8% cases.

Empyema of the gallbladder is often difficult to distinguish from uncomplicated acute cholecystitis [12]. In this study, 3 (1.3%) cases were reported as acute cholecystitis with empyema of gallbladder. This is in stark contrast to 31.5% cases of empyema associated with cholecystitis as reported by Memon [11]. Mucocele of the gallbladder has an incidence of 3 percent. In this study, 3.2 cases presented as cholecystitis with mucocele; reported incidence of mucocele is, however, several times higher [13,14].

Gallbladder polyps have an incidence ranging from 4.6 to 6.9 per cent [15]. In our study, one case of gallbladder polyp was identified. This low incidence can be attributed to small number of cases in our series. The only polyp discovered in our study was in a female. However, the prevalence of this pathology is much higher amongst males [16].

In our series, incidental carcinoma of gallbladder was found in 6 cases (2.7%). These gallbladders showed no gross abnormality per-operatively. The incidence of gallbladder malignancy in this series was considerably low compared to other studies, which show an incidence varying from 6.9 to 12 per cent [6,17,18]. The low incidence of malignancy in our series can be attributed to high sensitivity to exclusion criteria, where all patients with any preoperative evidence of malignancy, no matter how trivial, were excluded from the study. Low incidence of malignancy in our patients can also be attributed to increased acceptance and early reporting for laparoscopic cholecystectomy due to resort to day-care surgery at our institution. Samad [3] reports an incidence of 1.1% of malignancy in patients who underwent cholecystectomy for presumed chronic cholecystitis with cholelithiasis.

All patients in our series presented with longstanding history of chronic cholecystitis. There were no symptoms or signs suggestive of underlying malignancy in any patient; gallbladder malignancy usually does not have any characteristic clinical features with over 90 per cent of patients presenting with symptoms of acute or chronic cholecystitis [19]. Although ultrasound has a high diagnostic accuracy for both advanced and early gallbladder cancer [20], none of the six carcinomas in this series were picked on preoperative ultrasound. In addition, all six gallbladder specimens showed no macroscopic evidence of malignancy when they were opened during surgery. This is in contrast to the study by De Zoyasa et al. in whom all four cancers were suspected either on preoperative ultrasound or grossly during surgery; they suggest a more selective approach to gallbladder histology which may have saved both time and cost without having any unfavorable effects on patients well-being [21]. Similar observations and recommendations are made by other studies [22,23]. The issue of routine histopathology of all gallbladder specimen therefore remains unresolved; the need to send every specimen for histopathology or otherwise therefore depends on the expertise of the ultrasonologist as it depends on the skill of the operating surgeon. We, however, advocate routine histopathology of all gallbladders removed at surgery since the subsequent report would provide evidence of malignancy on solid grounds.

Although there are myriad of premalignant conditions, carcinoma gallbladder has a strong association with gallstones [17]. The strong association between the two warrants attention paid to histopathology of specimen in all cases undergoing cholecystectomy for cholelithiasis, irrespective of presence or otherwise of any gross abnormalities. It is widely reported that long standing mucosal irritation by the stones cause atypical cellular changes and increased cellular proliferation. It has been hypothesized that in long standing cases, these areas of hyperplasia progress to metaplasia and carcinoma-in- situ [24]. Studies confirm presence of such changes in the vicinity of gallbladder carcinoma [25]. The incidence of malignancy in our study was 2.7%, which is similar to that reported by Khan AH [26].

Histopathology revealed adenocarcinoma of gallbladder in six patients. One tumor was in stage T1b, two in T2 while one was in stage T3. While Cholecystectomy is an adequate treatment for pT1 tumors, pT2 and pT3 tumors require revision surgery to achieve a tumor-free surgical margin, along with lymph node dissection [27]. Three patients with the unexpected gallbladder cancer in stages T2 and T3 underwent a second standard revision procedure including transection of liver segments 4b and 5 (T2) and right hepatectomy (T3) with lymphadenectomy. These patients were followed up the oncological department for a period varying from 6 months to one year before being lost to follow up.

All six cases of incidentally diagnosed malignancies had associated gallstones, thereby strongly supporting the role of chronic irritation by long standing gallstones as etiological factor for carcinoma gall bladder.

Grading of cancer was found well differentiated to poorly differentiated adenocarcinoma, no case of squamous cell carcinoma, or other variant of cancerous histopathology seen.

## Conclusion

The histopathological spectrum of gallbladder after cholecystectomy is extremely variable. Incidental diagnosis of carcinoma gallbladder is not rare; we discovered evidence of malignancy in 6 (2.8%) cases on subsequent histopathological examination of gallbladder specimen, which showed no gross features of cancer. These cases had no symptoms suggestive of underlying malignancy nor was cancer reported on any of the preoperative investigations. Had the protocol of routine histopathology of all gallbladder specimens not been followed, these cases would have been failed to identify with disastrous results; We therefore, strongly advocate routine histopathology of all cholecystectomy specimens. Old patients and patients having long duration symptoms are strong candidates for histopathology of gallbladder specimen. The limitations of this study included a small sample size.

## Competing interests

The authors declare that they have no competing interests.

## Authors’ contributions

FGS carried out acquisition, analysis, interpretation of the data and drafting of the manuscript. AAM was involved interpretation of the data, drafting of the manuscript, and revised it critically for the intellectual content till the final version was reached. AHA, NAS and LA have read, edited and approved the final manuscript. All authors read and approved the final manuscript.

## Pre-publication history

The pre-publication history for this paper can be accessed here:

http://www.biomedcentral.com/1471-2482/13/26/prepub
